# Mobile App-Based Intervention for Pregnant Women With Stress Urinary Incontinence: Protocol for a Hybrid Effectiveness-Implementation Trial

**DOI:** 10.2196/22771

**Published:** 2021-03-10

**Authors:** Tiantian Li, Xiaomin Chen, Jia Wang, Ling Chen, Wenzhi Cai

**Affiliations:** 1 Department of Nursing Shenzhen Hospital Southern Medical University Shenzhen China; 2 School of Nursing Southern Medical University Guangzhou China

**Keywords:** mHealth, stress urinary incontinence, pregnancy, randomized controlled trial, process evaluation, mixed methods, study protocol

## Abstract

**Background:**

Stress urinary incontinence (SUI) is a common source of distress among women during and after pregnancy. It has a negative effect on quality of life but with poor care-seeking. Mobile health (mHealth) may be a promising solution with potential advantages. However, there is uncertainty whether a mobile app is effective for SUI symptom improvement during and after pregnancy. The implementation is also unclear. We developed an app named UIW (Urinary Incontinence for Women) aimed at improving perinatal incontinence.

**Objective:**

The objective of this study is to evaluate the effectiveness of the UIW app-based intervention in improving SUI symptoms among pregnant women and explore the facilitators and barriers to using the UIW app to help refine and optimize the intervention.

**Methods:**

This study is a hybrid effectiveness-implementation trial with a randomized controlled trial alongside a mixed-methods process evaluation according to the Reach, Effectiveness, Adoption, Implementation, and Maintenance (RE-AIM) framework. Pregnant women with SUI (n=336) will be recruited from a university-affiliated hospital in China. They will be randomly allocated (1:1) to either the intervention group that receive usual care plus UIW app or control group that receive usual care alone. The intervention period will last 2 months. The 5 dimensions of the RE-AIM framework will be evaluated at recruitment (-T1), baseline (T0), immediately after intervention (T1), 42 days after delivery (T2), 3 months after delivery (T3), and 6 months after delivery (T4) through project documents, online questionnaires and a pelvic floor muscle training diary, surface electromyography, log data in the background management system, and qualitative interviews. Data analysis will follow the intention-to-treat principle. Descriptive statistics, *t* tests, chi-square tests, and a linear mixed model will be used to analyze the quantitative data. Deductive and inductive content analysis will be used to analyze the qualitative data.

**Results:**

The effectiveness-implementation trial started in June 2020, trial recruitment was completed in October 2020, and the intervention will last for a 2-month period. Completion of the 6-month follow-up will be in July 2021, and we anticipate that the results of this study will be published in December 2021.

**Conclusions:**

This study will evaluate both effectiveness and implementation of the UIW app-based intervention among pregnant women. The hybrid effectiveness-implementation trial design according to the RE-AIM framework with a mixed-methods approach will give valuable insights into the effects as well as facilitators and barriers to the implementation that will influence the effects of the UIW app-based intervention.

**Trial Registration:**

Chinese Clinical Trial Registry ChiCTR1800016171; http://www.chictr.org.cn/showproj.aspx?proj=27455

**International Registered Report Identifier (IRRID):**

PRR1-10.2196/22771

## Introduction

### Background

Stress urinary incontinence (SUI) is the most prevalent type of urinary incontinence (UI) accounting for half of women suffering from UI [1,2]. SUI is defined as the complaint of involuntary urine leakage on effort or physical exertion (eg, sports activities) or during sneezing or coughing [3]. Pregnancy and childbirth are regarded as the major predisposing factors of SUI [4]. About 40% of women experience SUI during pregnancy [5], and the prevalence of SUI after delivery is over one-fifth of women [6]. Other types include urgency UI, which is characterized by involuntary urine leakage with a strong desire to urinate, and mixed urinary incontinence, which is the combination of SUI and urgency UI [7]. UI negatively affects women’s activities of daily life, mental well-being, and quality of life [8-10].

Pelvic floor muscle training (PFMT) is the first-line therapy for SUI, and the National Institute for Health and Care Excellence recommends women perform PFMT, especially during pregnancy [11]. Despite the availability of this effective therapeutic option for SUI, less than 30% of women seek help from health care professionals [12,13]. The reasons for not seeking treatment are multifaceted, such as embarrassment, stigma, insufficient knowledge, and perception of SUI among pregnant women [14]. Besides, a shortage of obstetric health care professionals and time restrictions during appointments also impede the delivery of perinatal incontinence care [15]. Given the unoptimistic condition, effective strategies are urgently needed to overcome these barriers. With the rapidly evolving development of information and communication technologies, mobile health (mHealth) may be a novel and promising strategy to improve perinatal SUI care [16].

mHealth is defined as achieving health objectives using mobile devices, such as mobile phones and other wireless devices [17]. With the advantages of anonymity, convenience, flexibility, and information support of mHealth, barriers to delivering perinatal incontinence care are lowered [18]. Women feel like they have greater privacy, are less embarrassed, have greater self-awareness of SUI, and appreciate the easy access to treatment options for SUI [19,20]. Moreover, 89% of the Chinese population use mobile phones, with a high prevalence of mobile internet usage in 2019 [21]. Thus, mHealth offers enormous potential to deliver SUI care to women on account of its advantages and high accessibility [16].

Several studies have proven mHealth to be effective for SUI management among community-dwelling women [22,23]. However, studies investigating the utility for mHealth to improve SUI symptoms during and after pregnancy are scarce [24]. Only one study was conducted with primiparas women [25]. Regretfully, it showed no significant difference in SUI symptoms between the mobile app–based intervention group and control group, and the reasons for the less-than-optimistic results were underexplained. The most recent Cochrane systematic review also revealed that the effect of antenatal PFMT as a treatment for UI in incontinent women is uncertain [26], which arose from poor reporting of the control arm and process details in the study [27]. Moreover, pregnant women’s use patterns and facilitators and barriers to using the app, referred to as process evaluation, should be explored further, which may explain variations in the success of mHealth interventions [28,29].

We developed a mobile app named UIW (Urinary Incontinence for Women) through an iterative and user-centered approach targeting pregnant women to improve perinatal incontinence care. It is an evidence-based and theory-driven app containing multiple intervention components based on the input of important stakeholders’ perspectives (pregnant women, obstetricians, and nurses). Therefore, we will assess the effectiveness and implementation of the UIW app, as adopted by pregnant women, compared to usual care, in this study, using a hybrid effectiveness-implementation study design through mixed-methods research [30,31]. The results of this study will also contribute to updating the Cochrane review to determine the effect of antenatal PFMT as a treatment for UI.

### Objectives

The overall goal of this effectiveness-implementation study is to provide more valuable insights into underlying reasons for the effective or ineffective results of app-based interventions among pregnant women with SUI compared to usual care. Then, it will provide a possibility to foster the implementation of an app-based intervention to improve incontinence care during and after pregnancy. There are 2 specific objectives in this study. First, we will evaluate whether the UIW app-based intervention is effective in improving SUI symptoms of pregnant women, as compared to usual care. Second, we will explore the facilitators and barriers to using the app during the implementation to help refine and optimize the intervention.

## Methods

### Study Design

**Figure 1:**
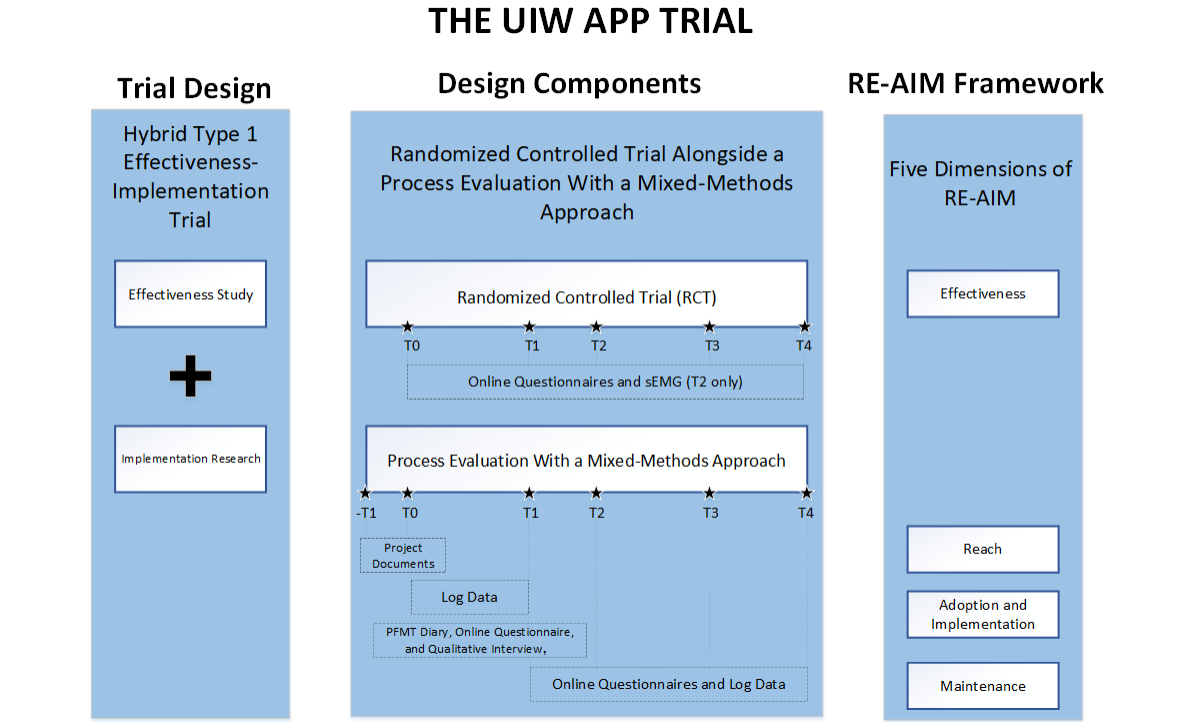
An overview of the trial design for the UIW (Urinary Incontinence for Women) app trial. PFMT: pelvic floor muscle training; RE-AIM: Reach, Effectiveness, Adoption, Implementation, and Maintenance; sEMG: surface electromyography; -T1: recruitment period; T0: baseline; T1: immediately after intervention; T2: 42 days after delivery; T3: 3 months after delivery; T4: 6 months after delivery.

This study is a hybrid type 1 effectiveness-implementation trial due to the need to determine the effectiveness of the UIW app-based intervention and understand the implementation to explain the effects of the intervention [30,31]. A randomized controlled trial (RCT) was designed to evaluate the effectiveness of the UIW app-based intervention compared to usual care among pregnant women with SUI, in parallel with an implementation research through a mixed-methods process evaluation following the explanatory sequential mixed-methods design (Figure 1) [32,33]. Reach, Effectiveness, Adoption, Implementation and Maintenance (RE-AIM) framework will be used to guide the evaluation, which is the vital evaluation theory on health promotion intervention [34,35].

**Figure 2:**
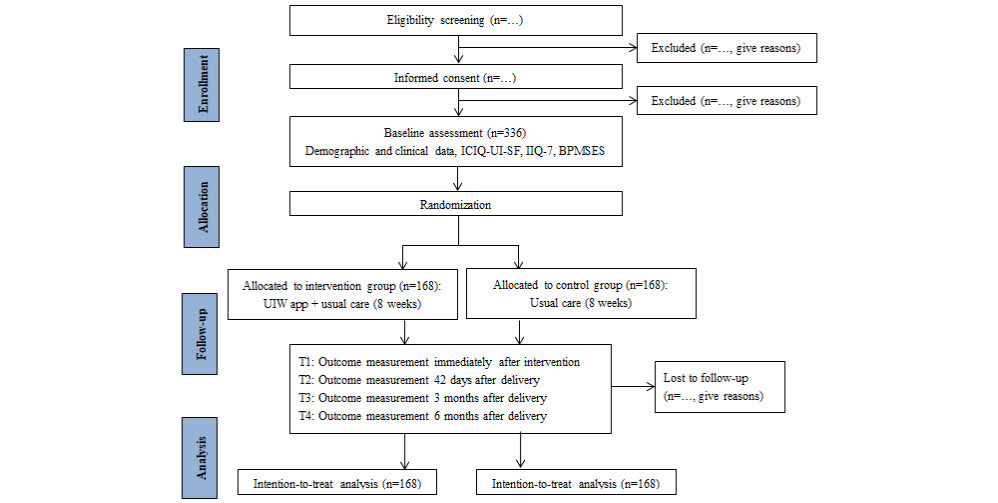
Trial flowchart according to CONSORT (Consolidated Standards of Reporting Trials). BPMSES: Broome Pelvic Muscle Self-Efficacy Scale; ICIQ-UI-SF: International Consultation on Incontinence Questionnaire-Urinary Incontinence Short Form; IIQ-7: Incontinence Impact Questionnaire-7; UIW: Urinary Incontinence for Women; T1: immediately after intervention; T2: 42 days after delivery; T3: 3 months after delivery; T4: 6 months after delivery .

This trial protocol has been developed in line with the Consolidated Standards of Reporting Trials of Electronic and Mobile HEalth Applications and onLine TeleHealth (CONSORT-EHEALTH) guidelines (Multimedia Appendix 1) [36], Standard Protocol Items for Randomized Trials (SPIRIT) 2013 statement (Multimedia Appendix 2) [37], and guidance for the qualitative research undertaken with the trial (Multimedia Appendix 3) [38]. The CONSORT flowchart is displayed in Figure 2.

### Setting

The study will be conducted in Shenzhen Hospital, Southern Medical University, a university-affiliated, tertiary level-A, public hospital in China. An average of 2000 pregnant women per month receive routine antenatal assessments in the hospital, and a vast majority of these pregnant women have access to a mobile phone and the internet [21].

### Eligibility of Participants

#### Inclusion Criteria

Pregnant women will be eligible if they are at least 18 years of age, have a singleton pregnancy according to ultrasonographic evaluation, are at 24-28 gestational weeks, have access to a mobile phone and the internet, and have SUI symptoms with ≥1 leakage episodes over the past 4 weeks (SUI symptoms are assessed by asking the question “When does urine leak?”). The question is an unscored item in the International Consultation on Incontinence Questionnaire-Urinary Incontinence Short Form (ICIQ-UI-SF), which has good reliability and validity [39]. SUI is defined as involuntary urine leakage on effort or physical exertion (eg, sports activities) or during sneezing or coughing [3]).

#### Exclusion Criteria

Pregnant women will be excluded if they have a psychiatric illness or cognitive impairment; previous UI; pelvic organ prolapse or pelvic surgery; or serious comorbidities or complications like heart disease, diabetes mellitus, hypertensive disorder of pregnancy, threatened abortion, placenta previa, placental abruption and premature rupture of membranes, fetal growth restriction, and amniotic fluid abnormalities.

### Recruitment and Informed Consent

Eligibility will be assessed by the researchers (TTL and JW) by checking the medical records from the obstetrics clinic. The researchers (TTL and JW) will introduce the study verbally, provide an information letter about the study with contact details of the researchers to eligible pregnant women, and patiently answer their questions. Pregnant women have 3 days to consider if they want to participate in this study. After agreeing, written informed consent will be obtained from participants by the researchers (TTL and JW), and a baseline electronic questionnaire including demographic and clinical data will be completed at the same time. The pregnant women will be informed that if there is any discomfort or pain associated with PFMT, they should contact the researchers by telephone without delay. The duration of enrollment is expected to cover a 3-month period. Pregnant women will be rewarded with a cash coupon (equal to RMB 150; US $23.19) after completing the study.

### Randomization and Blinding

The recruited pregnant women will be randomly assigned to either the intervention or control group with a 1:1 ratio using a simple randomization method. An independent research assistant will generate a random allocation sequence of 168 unique numbers using a random number table and write on different colored paper (pink paper for the first 168 numbers arranged in ascending order, white paper for the remaining numbers). This paper will be folded and put into consecutively numbered, sealed, opaque envelopes by the independent research assistant to conceal the allocations. The researchers (TTL and JW) will open the envelopes in numerical order during the process of recruiting and assigning participants. The participants with a pink paper will be assigned to the intervention group; others with a white paper will be assigned to the control group. The pregnant women in the intervention group will receive usual care plus the UIW app. The UIW app will be downloaded by scanning the QR code. The pregnant women in the control group will receive usual care alone.

Due to the nature of the app-based intervention and self-reported outcomes, except the assessment of pelvic floor muscle strength using surface electromyography (sEMG), it is not feasible to blind the participants or most of the outcome assessments. The experienced obstetricians delivering usual care and performing the sEMG tests are independent from this study and have no conflict of interest. They provide all participants with the same care and will remain blinded to the group allocation.

### Intervention

#### UIW App

**Figure 3:**
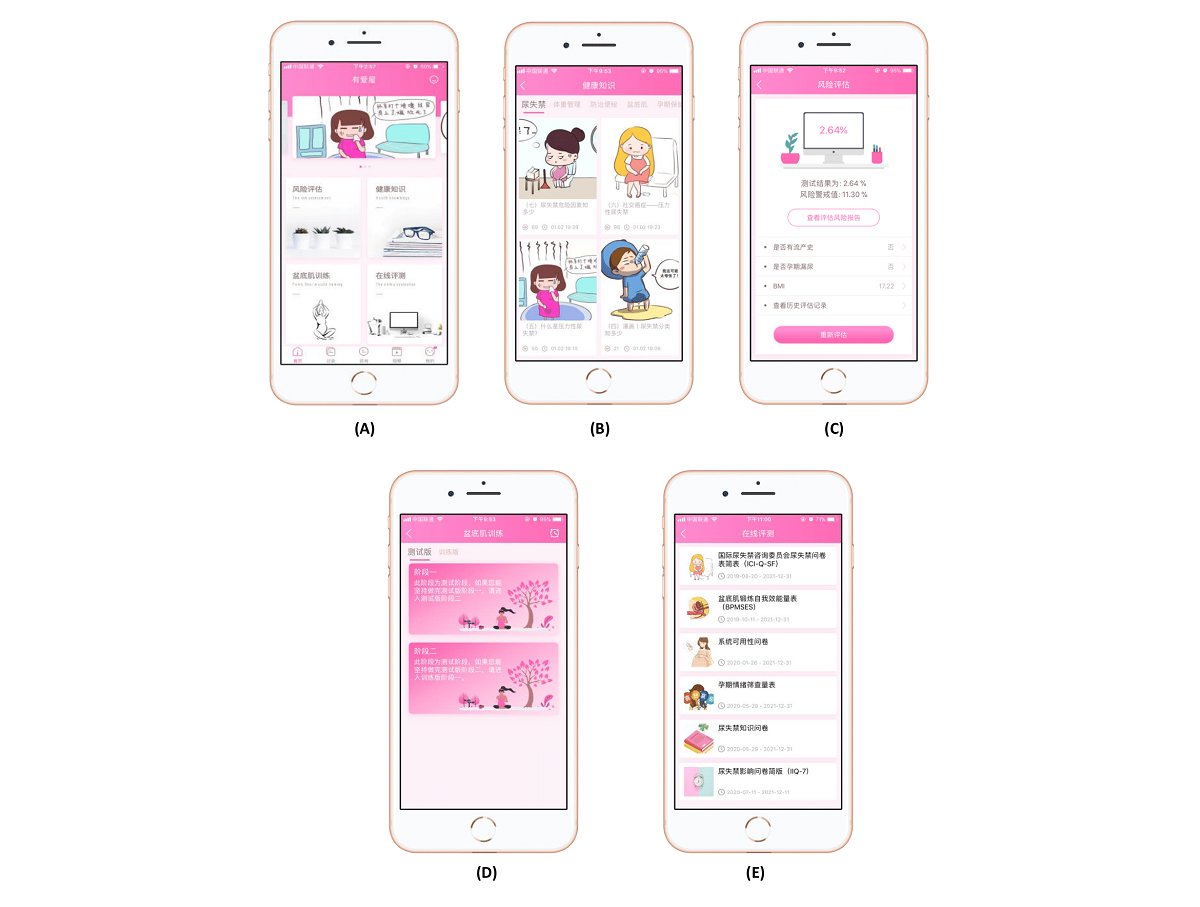
Screenshots of the UIW (Urinary Incontinence for Women) app, including the (A) Home Page, (B) Risk Assessment forum, (C) Health Education forum, (D) Pelvic Floor Muscle Training (PFMT) forum, and (E) Online Evaluation forum.

The UIW app is a mobile app for Chinese pregnant women with SUI developed by a research team from Shenzhen Hospital, Southern Medical University with technical assistance from the Guangdong Zhuoshang Network Technology Company (Registration Number: 2019SR1342273). There are 4 major modules in the UIW app including the Risk Assessment forum, Health Knowledge forum, PFMT forum, and Online Evaluation forum, with the functions of education, reminders, self-monitoring, and others, all arising from behavior change techniques (Multimedia Appendix 4) [40]. The 4 modules are visibly placed on the home page of the UIW app (Figure 3).

The Risk Assessment forum provides a risk prediction for SUI during the early stage of pregnancy based on a predictive tool previously developed by the research team [41]. According to the pregnant woman’s clinical risk factors, it identifies the high-risk population and gives feedback to the users whether they are at a high or low risk level.

The Health Education forum provides 5 topics specific to SUI and general pregnancy care, including SUI and lifestyle factors associated with SUI (weight management and constipation prevention [42,43], pelvic floor muscle care, and general antenatal care). A literature review, user needs analysis, and multidisciplinary expert group meeting were carried out to create the 5 topics.

The PFMT forum provides a personalized and instructional PFMT protocol adapted from the “Tät-an internet-based intervention providing PFMT to treat SUI” [44]. The PFMT is ordered by increasing difficulty. It consists of 2 basic levels and 4 advanced levels including different combinations and repetitions of 4 commonly used contraction types: test contraction, strength contraction, endurance contraction, quick contraction. When performing PFMT, the app shows real-time, dynamic guidance in the form of columnar graphics that present the duration and intensity of pelvic floor muscle contraction with concomitant relaxation. Pregnant women follow the image and sound command to contract and relax the pelvic floor muscles. Alarms can also be set to push notifications reminding the pregnant women to train.

The Online Evaluation forum provides reliable and validated questionnaires to evaluate the women’s SUI severity, quality of life, and self-efficacy with PFMT.

Additionally, at the bottom of the home page, there are other functions including recording of behaviors in daily life, consulting health care professionals about SUI-related questions, watching videos related to SUI, managing personal information, and system setup. Driven by administrative and adherence-focused guidance, the app also provides technical support, adherence monitoring, and reminders sent by the background management system in case the UIW is not logged into within 7 days [45]. The intervention period will last 2 months because research evidence indicates that 8 weeks PFMT is enough to improve the symptoms of SUI [46]. During the intervention period, pregnant women will autonomously use the app; it is up to them to determine their usage pattern in terms of frequency and duration of use.

The researcher will assist pregnant women in the intervention group with downloading the UIW app, registering, and installing their accounts. The pregnant women will be free to set up their usernames and passwords to submit the account application. Once the researcher confirms submission through the background management system, their accounts will be activated. The pregnant women will need to complete their personal information. Participants will be informed that the researchers have access to their profile.

A link to the electronic user manual and instructions for the UIW app will be fixedly displayed at the top of the home page. Technical support can be provided via the user manual and instructions on how to operate the UIW app. Moreover, the pregnant women can post their technology-related questions through the Assistance and Feedback function below the System Setup module. The researcher will respond to them within 24 hours.

#### Usual Care

The pregnant women in the intervention group and control group will both receive the same usual care. Usual care involves one-to-one health education about SUI and PFMT practice guidance at the time of recruitment by experienced obstetricians. It is delivered based on the Guidelines on the Medical Service Capacity of Tertiary Obstetrics and Gynecology Hospitals (National Health Commission of the People’s Republic of China, 2017 edition) [47]. The health education is verbally provided, which covers information corresponding to the 5 topics in the Health Education forum of the UIW app. PFMT practice guidance is teaching the correct pelvic floor muscle contraction technique and confirmation by obstetricians of a correct contraction through perineum palpation with the pregnant women in the supine position. Women will be directed to be aware of the feeling of selectively contracting muscles surrounding the urethra, vagina, and anus while relaxing the abdomen and buttocks muscles. Meanwhile, obstetricians will put one hand on the perineal body and the other hand on the abdomen to identify if women can correctly contract and relax the pelvic floor muscles; participants will be encouraged to contract the pelvic floor muscles more than 100 times per day [26].

### Measurements and Instruments of Data Collection

#### Overview

We will use a mixed-methods approach to evaluate the effectiveness and implementation of the trial according to the RE-AIM framework. Quantitative methods will be specifically used to measure the dimensions of Reach, Effectiveness, Adoption (UIW app usage results and patterns), Implementation, and Maintenance. For the Adoption dimension, qualitative methods will be used to explore the facilitators and barriers to adoption of the UIW app-based intervention from the pregnant women’s perspective. Multimedia Appendix 5 demonstrates the measures used in this study. Data will be collected at recruitment (–T1), baseline (T0), immediately after the intervention (T1), 42 days after delivery (T2), 3 months after delivery (T3), and 6 months after delivery (T4). Data will be collected from both the UIW app-based intervention group and control group. For the Reach dimension, the participation rate of eligible pregnant women will be examined using the project document data recorded by the researchers. For the Effectiveness dimension, there is 1 primary outcome, and there are 4 secondary outcomes, which are described in the following sections.

#### Primary Outcome

Symptoms of SUI will be evaluated using the ICIQ-UI-SF [48]. The ICIQ-UI-SF is a 4-item instrument with 3 scored items assessing the frequency of leakage, amount of leakage, and overall impact of leakage on life and 1 unscored item diagnosing the type of UI. The total score ranges from 0 to 21, with 0-7 indicating mild symptoms, 8-13 indicating moderate symptoms, and 14-21 indicating severe symptoms. The Chinese version of the ICIQ-UI-SF has a Cronbach α coefficient of 0.71 and over 95% agreement between the 2 tests [49].

#### Secondary Outcomes

The secondary outcomes include pelvic floor muscle strength, quality of life, self-efficacy with PFMT, and risk factors for SUI.

Pelvic floor muscle strength will be assessed using sEMG with the Vishee neuro-muscle stimulator (MyoTrac Infiniti, model SA9800, Thought Technology Ltd, Montreal, Quebec) [50]. It will be performed by following the Glazer protocol by 2 independent and experienced obstetricians who have passed the medical qualification examination and have more than 5 years of working experience. After participants are in a supine lithotomy position, a drop-shaped vaginal probe (registration certificate number: 20152211142, type VET-A, produced by Nanjing Vishee Medical Technology Ltd, Nanjing China) will be placed into the vagina. Participants will be instructed how to correctly contract the pelvic floor muscles to avoid crosstalk contamination of the surrounding muscles before starting the test. Then, participants will follow the text hints and voice prompts of the automated protocol software to contract and relax the pelvic floor muscles. The whole test includes 5 indicators of amplitude (µV): pretest resting, phasic contraction, tonic contraction, endurance contraction, and posttest resting. A lower amplitude indicates weaker pelvic muscle strength. sEMG is a reliable way to measure pelvic floor muscle strength, and its values are closely related to SUI [51].

Quality of life among pregnant women with SUI will be assessed using the Incontinence Impact Questionnaire-7 (IIQ-7) [52]. The IIQ-7 is a 7-item instrument with 4 domains including physical activity, travel, social activities, and emotional health. Higher total score indicates a higher level of impact on life. The Chinese version of the IIQ-7 has a Cronbach α coefficient of 0.824 and high construct validity [53].

Self-efficacy with PFMT will be measured using the Broome Pelvic Muscle Self-Efficacy Scale (BPMSES) [54]. The BPMSES is a 23-item instrument with 14 items in the dimension of self-efficacy expectations and 9 items in the dimension of outcome expectations. A total score of 0-33 indicates low self-efficacy, 34-66 indicates moderate self-efficacy, and 66-100 indicates high self-efficacy. The Chinese version of the BPMSES has a Cronbach α coefficient of 0.912 and a test-retest reliability coefficient of 0.910 [55].

Risk factors for SUI among pregnant women will be assessed using a self-designed questionnaire, investigating weight, condition of constipation, active and passive smoking, and consumption of liquids containing caffeine.

For the dimension of Adoption, the pregnant women’s PFMT adherence and activity in the 2 groups, usage results and patterns of use of the UIW app for the intervention group, other possible apps and websites used by the control group, and facilitators and barriers to adoption of the app will be explored through quantitative and qualitative methods. PFMT adherence and activity as well as usage results and patterns will be evaluated using quantitative methods through the log data of the background management system for the intervention group; a self-administered, electronic PFMT diary; and an electronic questionnaire (Multimedia Appendix 5). For the qualitative interviews, we will adopt a purposive sampling strategy to select women from the 2 groups based on the primary outcome of the SUI symptom score at the end of the intervention. Pregnant women will be selected at a ratio of 1:1 in the higher and lower scores until achieving data saturation such that no new themes emerge during data analysis. The interviews will be performed by a researcher (TTL) via telephone or face-to-face. The interviewer is a female nurse with a master’s degree in nursing science and formal training in qualitative studies. A semistructured interview guide was developed by the research team and covers the topics of user experiences, preferences, and expectations (Multimedia Appendix 5). Data collection, management, and analysis will be carried out concurrently, which makes it possible to adapt and refine the interview guide [56]. All interviews were audiotaped after gaining permission from the participants.

For the dimension of Implementation, the fidelity of the intervention and usual care provided will be evaluated (Multimedia Appendix 5).

For the dimension of Maintenance, the measures used to assess “Effectiveness,” “Adoption,” and “Implementation” will be repeatedly assessed in the follow-up period at the T2, T3, and T4 time points.

### Data Management and Analysis

#### Quantitative Data

To avoid bias due to unnecessary face-to-face contact, data for pregnant women in both the intervention group and control group are collected electronically, using app-based and internet-based methods, respectively. After the data are exported from the app’s background management system and questionnaire website, data will be entered directly in a special database for the UIW app and saved in the database by researchers on a password-protected computer. Each participant is identified by a unique study ID assigned by the independent researcher assistant at the time of recruitment. And the “log” connecting the participants with the study IDs is held by the independent research assistant.

SPSS (version 25.0; IBM Corp, Armonk, NY) will be used for all quantitative data analysis. Descriptive statistics will be used to show continuous variables as mean (SD) and categorical variables as frequency and percentage. The baseline data will be compared between the intervention and control groups as well as between participants completing the follow-up of this study and those who drop out, with the independent sample *t* test for continuous variables and the chi-square test or Fisher exact test for categorical variables. The effects of the intervention within each group will be assessed with a paired *t* test for the severity of SUI, quality of life, and self-efficacy with PFMT and a McNemar chi-square test for risk factors. To compare the effects of the intervention between groups for the repeated measures, including severity of SUI, quality of life, and self-efficacy with PFMT, a linear mixed model will be used, which allows for missing data [57]. The model will include subjects as random effects and time, intervention, and the interaction between intervention and time as fixed effects. Pelvic floor muscle strength will be compared between groups using an independent sample *t* test. For the risk factors and PFMT adherence, chi-square tests will used to compare the differences between groups. *P* values <.05 will be considered statistically significant.

Participants with missing data will also be included in the analysis following the principle of intention-to-treat analysis. Missing data at follow-up will be replaced with the corresponding values at baseline.

#### Qualitative Data

The semistructured, in-depth interviews conducted by telephone or face-to-face will be transcribed verbatim into text by the researchers (TTL and JW) within 24 hours after the telephone interview. Qualitative data analyses will be performed using NVivo software (version 11; QSR International, Melbourne, Australia). We will apply deductive and inductive content analysis to analyze the transcripts. Two researchers will separately perform the initial coding. The codes will be checked for agreement, compared regarding the similarities and differences, and grouped into subcategories and categories to form subthemes and themes. Several techniques will used to improve the trustworthiness of the qualitative study, including respondent validation, triangulation, peer debriefings, and audit trails.

#### Sample Size Calculation

The sample size calculation was based on the primary outcome of the SUI symptom score, which is used to estimate the effect size in the power analysis [58]. A previous mobile app–based study performed among primiparas women showed a moderate effect size (0.344) [25]. G*Power [59] was used to estimate the sample size. A minimum sample size of 268 pregnant women is required for this study to achieve a power of 0.80 at a 2-sided significance level of .05. A 20% attrition rate is estimated based on previous studies involving mHealth interventions [60]. The total sample size was increased to 336, with 168 in each group.

### Ethical Approval

The study has received ethical approval from the Ethics Committees of Shenzhen Hospital, Southern Medical University (approval number NYSZYYEC20190012) on August 27, 2019. It will be performed in accordance with Helsinki 1964 and later revisions and overseen by the independent trial steering committee (TSC) and data monitoring and ethics committee (DMEC). The TSC provides overall supervision for this research on behalf of the funder, and the DMEC reviews confidential interim analyses semiannually to monitor data, oversee trial safety and progress, and make recommendations. In addition, all participants in this study will provide written informed consent and hold rights to withdraw from this trial in anytime. Participants’ data will be anonymous and stored on a password-protected background management system. Any adverse events experienced by participants during this study will be recorded and reported to the DMEC.

## Results

The effectiveness-implementation trial started in June 2020, trial recruitment was completed in October 2020, and the intervention will last for a 2-month period. Completion of the 6-month follow-up will occur in July 2021, and we anticipate that the results of this study will be published in December 2021.

## Discussion

Research has indicated that a vast majority of pregnant women are expected to screen for SUI [61]. mHealth is a promising solution to improve SUI care during and after pregnancy. This study will provide valuable information on the effectiveness and implementation of the UIW app-based intervention aimed at improving SUI symptoms in women during and after pregnancy. By using a hybrid type 1 effectiveness-implementation trial, an extensive process evaluation with a mixed-methods approach will be performed alongside an RCT. Facilitators and barriers to the use of the UIW app-based intervention will also be explored. The results will provide a deep understanding of the underlying effect mechanism of the app-based intervention. This will contribute to better implementation and dissemination in the clinical context.

Previous studies were mostly aimed at community-dwelling women [22,23]. Only 1 study was performed among pregnant women but did not show significant effects [25]. The underlying reasons are unclear. To the best of our knowledge, this is the first study to evaluate an app-based intervention for SUI distress in pregnant women using a hybrid type 1 effectiveness-implementation trial design. If the UIW app-based intervention is effective, this study will provide evidence to improve perinatal incontinence care using a mobile app.

### Strengths and Limitations

This study is powered by some strengths. First, the UIW app functions are comprehensive. It not only focuses on PFMT but also merges various behavior change techniques, including shaping knowledge, feedback, self-monitoring, and others [40]. Second, given the implementation of “Two-Child Policy” in China, the number of multiparas women is obviously increasing [62]. To evaluate the effectiveness of the UIW app-based intervention in a more realistic clinical context, we will enroll both primiparas and multiparas women in this study. Third, a hybrid type 1 effectiveness-implementation design, with RE-AIM as the guidance framework, is applied in this study. The RE-AIM framework specifies multiple domains of evaluation [34] and has been commonly utilized in research on health promotion intervention [63]. Knowledge gained from this sophisticated design will make a great contribution to the RCT evaluating the mHealth intervention. Furthermore, integration of quantitative and qualitative methods will occur at various levels through the mixed-methods design of the implementation evaluation [32]. At the study design level, an explanatory sequential design is employed. In the qualitative study, interview samples will be purposely selected from the participants of the RCT according to the primary outcome. Thus, integration occurs through connecting and building at the methods level.

There are several potential limitations. One limitation may be that the sEMG will be only assessed at 42 days after delivery. Considering that an sEMG is conducted via a probe placed into the vagina, we will not assess sEMG during pregnancy due to the potential risk. Participants will seldomly return to the hospital after the reexamination at 42 days after delivery, and it is difficult to reach the participants. In view of feasibility and acceptability, the sEMG assessments will not also be conducted at 3 months and 6 months after delivery. Another limitation may lie in the requirements of having a mobile phone and access to the internet. It may lead to a more tech-savvy population who are more familiar and comfortable with mobile phone use and limit the generalizability to the whole population of pregnant women, although the rates of internet use and mobile phone ownership are relatively high. Finally, though the process evaluation is important to interpret the trial findings, it aims to provide transparency of the implementation of the intervention [64]. It only provides a possible explanation about the results rather than a full explanation.

### Conclusions

Although mHealth is a promising technology, the effectiveness and implementation of mHealth interventions need to be explored. The UIW app is an evidence-based and theory-driven app containing a comprehensive set of functions. In this study, we will investigate the effectiveness of as well as facilitators and barriers to the implementation of the UIW app-based intervention among pregnant women. A hybrid effectiveness-implementation trial design according to the RE-AIM framework has been adopted, with an RCT conducted in parallel with a mixed-methods process evaluation. This study will expand the understanding of the app-based intervention to improve the SUI situation among pregnant women. Insights into the study results can enhance the implementation and dissemination of this intervention in the clinical context.

## Appendix

Multimedia Appendix 1 CONSORT-eHEALTH checklist (V 1.6.1).

Multimedia Appendix 2 SPIRIT 2013 checklist.

Multimedia Appendix 3 Guidance for the qualitative research undertaken with trial.

Multimedia Appendix 4 Mapping of the Behavior Change Techniques and elements in UIW app UIW: Urinary Incontinence for Women; PFMT: Pelvic Floor Muscle Training.

Multimedia Appendix 5 List of measures. -T1: recruitment period; T0: baseline; T1: immediately after intervention; T2: 42 days after delivery; T3: 3 months after delivery; T4: 6 months after delivery.

## Abbreviations

BPMSES: Broome Pelvic Muscle Self-Efficacy Scale
CONSORT-EHEALTH: Consolidated Standards of Reporting Trials of Electronic and Mobile HEalth Applications and onLine TeleHealth
DMEC: data monitoring and ethics committee
ICIQ-UI-SF: International Consultation on Incontinence Questionnaire-Urinary Incontinence Short Form
IIQ-7: Incontinence Impact Questionnaire-7
mHealth: mobile health
PFMT: pelvic floor muscle training
RCT: randomized controlled trial
RE-AIM: Reach, Effectiveness, Adoption, Implementation and Maintenance
sEMG: surface electromyography
SPIRIT: Standard Protocol Items for Randomized Trials
SUI: stress urinary incontinence
TSC: trial steering committee
UI: urinary incontinence
UIW: urinary incontinence for women
