# Supplementary material for: Mobile App-Based Intervention for Pregnant Women With Stress Urinary Incontinence: Protocol for a Hybrid Effectiveness-Implementation Trial
Source: JMIR Res Protoc. 2021 Mar 10;10(3):e22771. doi: 10.2196/22771 (PMC7991980; doi:10.2196/22771)
Supplement: Multimedia Appendix 3 [file resprot_v10i3e22771_app3.pdf]

Multimedia Appendix 3 Guidance for the qualitative research undertaken with trial

Item	Item No	Description	Reported on page number
Aim	1	Describe the aim of the qualitative research. Where appropriate identify aims specific to the trial e.g. ‘to explore patient views on adherence to the trial intervention’ rather than using general aims e.g. ‘to explore patient experiences’.	Page 2
Rationale	2	Describe the rationale for including qualitative research; identify areas of uncertainty to be explored. Include a statement addressing the ways in which the aims of the qualitative research will ‘add value’ to the trial.	Page 2 and 8
Methods	3a	Provide a clear account of the proposed methods of data collection including the location and timing of data collection, and the skills and seniority of the person who will undertake data collection.	Page 7 and Multimedia Appendix 5
	3b	Describe the sample frame, sampling method(s), and sample size. Where the sample frame is trial participants, specify whether intervention, control or both will be included.	Page 7
	3c	Describe and reference the proposed approach to analysis. A rationale for the approach to be taken may be included.	Page 7
	3d	Identify the qualitative research skills and seniority of the person who will undertake the analysis and write-up.	Page 7
Integration with trial	4	Outline suggestions for integrating and synthesising qualitative data / findings with the trial results.	Page 8
Costs	5	Describe the full costs of the qualitative research and highlight any dedicated equipment, software, staff, and transcription costs.	Page 7
Leader ship	6	Identify which of the co-applicants will take overall responsibility for the qualitative research and describe their role in the design, data collection, analysis and write-up of the study.	Page 7 and 9
