# Supplementary material for: Mobile App-Based Intervention for Pregnant Women With Stress Urinary Incontinence: Protocol for a Hybrid Effectiveness-Implementation Trial
Source: JMIR Res Protoc. 2021 Mar 10;10(3):e22771. doi: 10.2196/22771 (PMC7991980; doi:10.2196/22771)
Supplement: Multimedia Appendix 4 [file resprot_v10i3e22771_app4.pdf]

Multimedia Appendix 4 Mapping of the Behavior Change Techniques and elements in UIW app UIW: Urinary Incontinence for Women; PFMT: Pelvic Floor Muscle Training.

Behavior Change Technique cluster	Behavior Change Technique components	Constitutive definition	UIW app elements generated
Goals and planning	Action planning	Immediate detailed planning for performing the behaviour	PFMT forum and alarms for exercise
Feedback and monitoring	Feedback on behaviour	Monitoring and feedback on performance of behaviour	Adherence monitoring and reminders sent by background management system
	Self-monitoring of behaviour	A method for person to record and monitor their behaviours.	Function of recording behaviour in daily life: e.g. uptake of liquid containing caffeine
	Self-monitoring of outcome(s) of behaviour	A method for person to record and monitor their behaviour outcomes	Online Evaluation forum and function of recording urine leakage in daily life
Social support	Social support (general)	Advise or social support from others for performing the behaviour, such as counselling	Function of consulting health care professionals
Shaping knowledge	Instruction on how to perform a behaviour	Advise on the method of performing the behaviour (includes 'skills training')	Health Education forum and the function of watching videos
Natural consequences	Information about health consequences	Health consequences-related Information of performing the behaviour or not	Risk Assessment forum , Health Education forum and the function of watching videos, e.g. worsening symptoms
	Information about social and environmental consequences	Social and environmental consequences-related Information of performing the behaviour or not	Health Education forum and the function of watching videos, e.g. reducing social activity, lowering quality of life

Comparison of behaviour	Modeling of the behaviour	An observable sample of how to perform the behaviour	Health Education forum and the function of watching videos
Associations	Prompts/cues	Stimulus defined to prompt or cue the behaviour	Alarms for exercise in PFMT forum
Repetition and substitution	Graded tasks	Easy-to-perform tasks set to make them increasingly difficult, but achievable, until the behaviour is achieved	PFMT protocol ordered by increasing difficulty in PFMT forum
Comparison of outcomes	Persuasive argument	Visual or verbal communication from credible sources	Health Education forum and function of consulting health care professionals, e.g. sources of literature and health professionals
