# Supplementary material for: Mobile App-Based Intervention for Pregnant Women With Stress Urinary Incontinence: Protocol for a Hybrid Effectiveness-Implementation Trial
Source: JMIR Res Protoc. 2021 Mar 10;10(3):e22771. doi: 10.2196/22771 (PMC7991980; doi:10.2196/22771)
Supplement: Multimedia Appendix 5 [file resprot_v10i3e22771_app5.pdf]

Multimedia Appendix 5 List of measures. -T1: recruitment period; T0: baseline; T1: immediately after intervention; T2: 42 days after delivery; T3: 3 months after delivery; T4: 6 months after delivery.

Aims	RE-AIM[a] dimensions	Definitions	Measures	Data sources	Data types	Time points administered -T1	T0	T1	T2	T3	T4
Effectiveness	Effectiveness	The effects of the UIW[b] app-based intervention to improve pregnant women's SUI[C] status compared to usual care	Primary outcomes:	Questionnaire and sEMG	Quan[i]						
			· Symptom of SUI: ICIQ-UI-SF[d]				√	√	√	√	√
			Secondary outcomes:		Quan						
			· Pelvic floor muscle strength: sEMG[e]						√		
			· Quality of life: IIQ-7[f]				√	√	√	√	√
			· Self-efficacy of PFMT[g]: BPMSES[h]				√	√	√	√	√
			· Risk factors of SUI: self-designed questionnaire				√	√	√	√	√
Implementation	Reach	Proportion of target population participating in the study	Participation rate: · Sample size divided by the approached eligible target population	Project documents	Quan	√					
	Adoption	The extent of UIW app modules and functions actively engaged by the participants of the intervention group and PFMT activity and other accessible apps and websites use for participants of the control group in the 2-month intervention period	Adherence of PFMT: · Actual PFMT dosage: Number of repetitions, duration frequency and frequency of PFMT for women. Pregnant women performing PFMT reach the dosage of three days a week or above are regarded as adherence, otherwise non-adherence.	Log data of background management system for intervention group and electronic PFMT diary and questionnaire for control group	Quan			√			
			App usage results and patterns: ·For the intervention group: Total and 2-month mean of frequency and duration of logins, actual activity of each module in UIW app ·For the control group: Self-reported use					√			

		frequency and duration of other accessible apps and websites and their names or sources			
	User experience and burden, expectation and preference of using the app	Facilitators and barriers to Adoption of the UIW app:<br>· A semi-structured topic guide: Focused on participants' perceptions and experience of the UIW app-based intervention based on the similar study, issues arising during the intervention period and research team discussion<br>· What is your experience of using the UIW app/other available apps or websites?<br>· What are the advantages of UIW app/ other available apps or websites you used?<br>· What are the disadvantages of UIW app/ other available apps or websites you used?<br>· Do you have any suggestions for improvement?<br>· Would you like to recommend the UIW app to others?(For intervention group) Would you like to use UIW app?(For control group) And why?	Qualitative interview	Qual[j]	√
Implementation	The extent of the trial protocol followed as planned	Intervention fidelity:<br>·UIW app:<br>· Total number and 2-month mean of late or lacking technical supports<br>· Total number and 2-month mean of late or	Log data of background management system and questionnaire	Quan	√

		lacking consultation responses<br>· Total number and 2-month mean of reminders sent to inactive users without login the app during the past seven days<br>· Usual care provided:<br>· If verbal health education has been delivered by obstetricians?<br>· If PFMT practice guidance and confirmation have been delivered by obstetricians?						
Maintenance	Degree to sustainability of the intervention and its effects	Measures are same as “Effectiveness”, “Adoption” and “Implementation” above	Questionnaire and log data of background management system	Quan		√	√	√

[a] RE-AIM: Reach, Effectiveness, Adoption, Implementation and Maintenance.

[b] UIW: Urinary Incontinence for Women.

[C] SUI: Stress Urinary Incontinence.

[d] ICIQ-UI-SF: International Consultation on Incontinence Questionnaire-Urinary Incontinence Short Form.

[e] sEMG: Surface Electromyography.

[f] IIQ-7: Incontinence Impact Questionnaire-7.

[g] PFMT: Pelvic Floor Muscle Training.

[h] BPMSES: Broome Pelvic Muscle Self-Efficacy Scale.

[I] Quan: Quantitative.

[j] Qual: Qualitative.
